# Supplementary material for: Piezoresistance Characterization of Silicon Nanowires in Uniaxial and Isostatic Pressure Variation
Source: Sensors (Basel). 2022 Aug 23;22(17):6340. doi: 10.3390/s22176340 (PMC9459738; doi:10.3390/s22176340)
Supplement: Supplementary file 1 [file sensors-22-06340-s001.zip › sensors-1846940-supplementary.pdf]

sensors MDPI

Supplementary Material

Piezoresistance Characterization of Silicon Nanowires in Uniaxial and Isostatic Pressure Variation

Elham Fakhri [1,*], Rodica Plugaru [2], Muhammad Taha Sultan [1,3], Thorsteinn Hanning Kristinsson [1], Hákon Örn Árnason [1], Neculai Plugaru [2], Andrei Manolescu [1], Snorri Ingvarsson [3] and Halldor Gudfinnur Svavarsson [1,*]

1 Department of Engineering, Reykjavik University, Menntavegur 1, 102 Reykjavik, Iceland
2 National Institute for Research and Development in Microtechnologies-IMT Bucharest, 077190 Voluntari, Romania
3 Science Institute, University of Iceland, Dunhaga 3, 107 Reykjavik, Iceland

check for updates

1. Periodic nanowires

In this Section we show the samples with periodic arrays of nanowires and the corresponding results, to be compared with the random wires presented in the main text. These samples were processed in 10 Ω cm p-type (100) Si substrates, by chemical etching method, using 1:1 H_20: HF 4 mL+0.1 mL H_2O_2 solution. Vertical nanowires with length/diameter of 650 nm/272 nm, as given by SEM investigations, resulted when the etching time was 5 min. In order to achieve the electrical contact to the SiNW arrays, Al_2O_3 (2%) doped ZnO films (about 43 nm thick and resistivity of about 210 Ω cm) were deposited on the samples by RF magnetron sputtering using an Oxford PlasmaLab System 400 equipment. The electrodes consist of copper wires connected to the SiNW arrays and also to Si substrate by silver conductive glue paste. SEM images of the periodic SiNWs are shown in Figure S1.

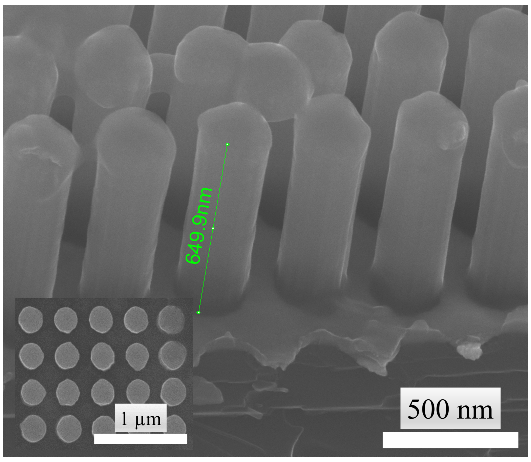

Figure S1. Cross-sectional SEM micrographs of periodic SiNWs-ZnO array with wire length of 650 nm, inset shows the top view image.

Isostatic pressure in the range 1 bar to 35 bar (0.1 MPa to 3.5 MPa) was applied on this sample by placing it in a controlled pressure chamber Type 4E/2lt, Büchiglasuster-Switzerland, under nitrogen atmosphere. The relative resistance variation $\Delta R/R_0$ was in the interval 6 % to 18 % at pressures between 0.5 MPa to 3.5 MPa at direct bias, as shown in Figure S2. The PZR had only a slight dependence on applied pressure for reversed bias (not shown).

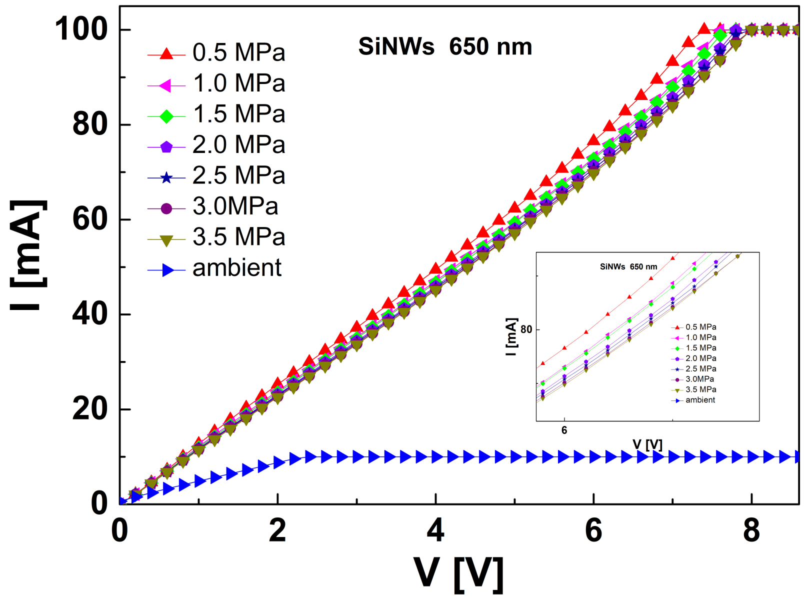

(**a**)

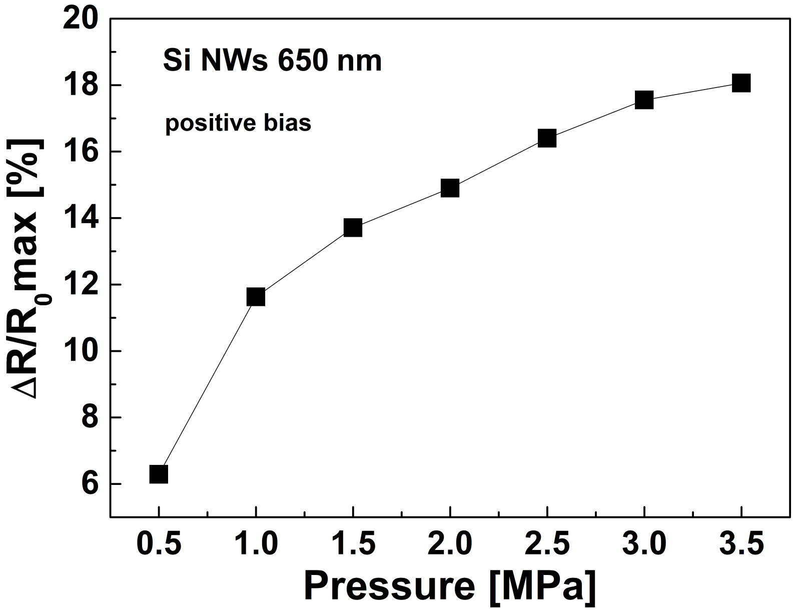

(**b**)

Figure S2. (**a**) I-V characteristics for direct bias of the 650 nm periodic SiNWs, with applied pressure in the range 0.5 MPa to 3.5 MPa. The blue curve corresponds to atmospheric pressure. (**b**) Variation of $\Delta R/R_0$ with applied pressure for a fixed direct bias of 2 V.
